# Deciphering the importance of the palindromic architecture of the immunoglobulin heavy-chain 3' regulatory region

**DOI:** 10.1038/ncomms10730

**Published:** 2016-02-17

**Authors:** Alexis Saintamand, Christelle Vincent-Fabert, Armand Garot, Pauline Rouaud, Zeliha Oruc, Virginie Magnone, Michel Cogné, Yves Denizot

**Affiliations:** 1 Université de Limoges, CRIBL, UMR CNRS 7276, Limoges 87025, France; 2 CNRS et Université de Nice Sophia Antipolis, Institut de Pharmacologie Moléculaire et Cellulaire, UMR 6097, Sophia Antipolis 06560, France; 3 Institut Universitaire de France, Paris 75231, France

## Abstract

The *IgH* 3' regulatory region (*3'RR*) controls class switch recombination (CSR) and somatic hypermutation (SHM) in B cells. The mouse *3'RR* contains four enhancer elements with *hs1,2* flanked by inverted repeated sequences and the centre of a 25-kb palindrome bounded by two *hs3* enhancer inverted copies (*hs3a* and *hs3b*). *hs4* lies downstream of the palindrome. In mammals, evolution maintained this unique palindromic arrangement, suggesting that it is functionally significant. Here we report that deconstructing the palindromic *IgH 3'RR* strongly affects its function even when enhancers are preserved. CSR and *IgH* transcription appear to be poorly dependent on the *3'RR* architecture and it is more or less preserved, provided *3'RR* enhancers are present. By contrast, a ‘palindromic effect' significantly lowers *V*_*H*_ germline transcription, AID recruitment and SHM. In conclusion, this work indicates that the *IgH 3'RR* does not simply pile up enhancer units but also optimally exposes them into a functional architecture of crucial importance.

## Abstract (web summary)

The *IgH* 3' regulatory region contains an evolutionarily conserved palindromic sequence flanking important enhancer elements. Here the authors show that the palindrome is required for generating antibody diversity.

## Graphical abstract

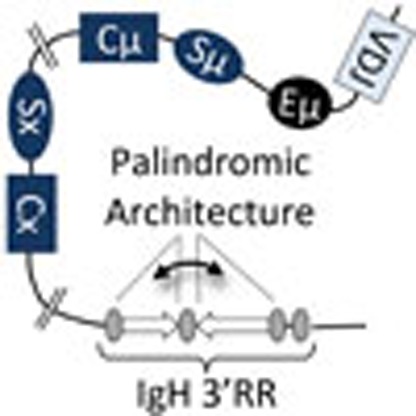

Lymphopoiesis is coupled with programmed accessibility of Ig genes to transcription and to several major transcription-dependent DNA-remodelling events^1^,^2^. Multiple *cis*-regulatory elements located 5' and 3' of constant (*C*) genes control B-cell ontogeny. Among 5' elements, the intronic *Eμ* enhancer is reported as a master control element of *V(D)J* recombination^3^,^4^. The *IgH* 3' regulatory region (*3'RR*), which encompasses the four transcriptional enhancers *hs3a, hs1,2*, *hs3b* and *hs4*, controls μ-transcription in mature B cells^5^, and is the master element controlling conventional class switch recombination (CSR)^6^,^7^, locus suicide recombination^8^ and somatic hypermutation (SHM)^9^ but with little role in *V(D)J* recombination, except for silencing early transcription in pro-B cells^10^,^11^,^12^. The mouse *3'RR* contains four enhancer elements (*hs3a*, *hs1,2*, *hs3b* and *hs4*) with *hs1,2* flanked by inverted repeated intervening sequences (*IRIS*) and the centre of a >25-kb palindrome bounded by 2 inverted copies of the *hs3* enhancers (*hs3a* and *hs3b*) refs 2, 13. *hs4* lies downstream of the palindrome. The modest activity of each of the *3'RR* elements *in vitro*, however, contributes to a synergic and potent global effect of the *3'RR* in transgenes, especially when its ‘palindromic' architecture is maintained^14^. In humans, each of the two *3'RR* located downstream of *Cα1* and *Cα2* contains three enhancer elements similar to mouse *hs3*, *hs1,2* and *hs4*, with *hs1,2* being also flanked by *IRIS*. Beyond divergence of *hs1,2*-flanking sequences, evolution maintained a ‘quasi-palindromic' organisation in all mammals for which sequence is available, making it tempting to speculate that this unique arrangement is of significant importance for the regulatory region function^15^. To explore the role of the *3'RR* palindromic architecture in the context of the endogenous locus, we analysed two newly generated transgenic mice: *hs3a*+*IRIS*+*hs1,2*-deficient mice (called ΔleftPAL mice in this study) lacking the 11.5-kb left half of the *3'RR* palindrome (deconstructing palindrome and deleting two enhancer elements) and Δ*IRIS* mice with the same left-half deletion of the palindrome but with reintroduction of a inverted *hs3a* and *hs1,2* enhancers (deconstructing the palindrome by fully eliminating left-side *IRIS* while maintaining all four core enhancer elements). We report that the deconstruction of the *IgH* 3' palindrome widely affects SHM but only marginally affects CSR, showing that the unique architecture of the *IgH* locus 3' boundary crucially determines the full functional expression of the *IgH 3'RR* transcriptional enhancers.

## Results

### Generation of ΔleftPAL and ΔIRIS mice

**Figure 1:**
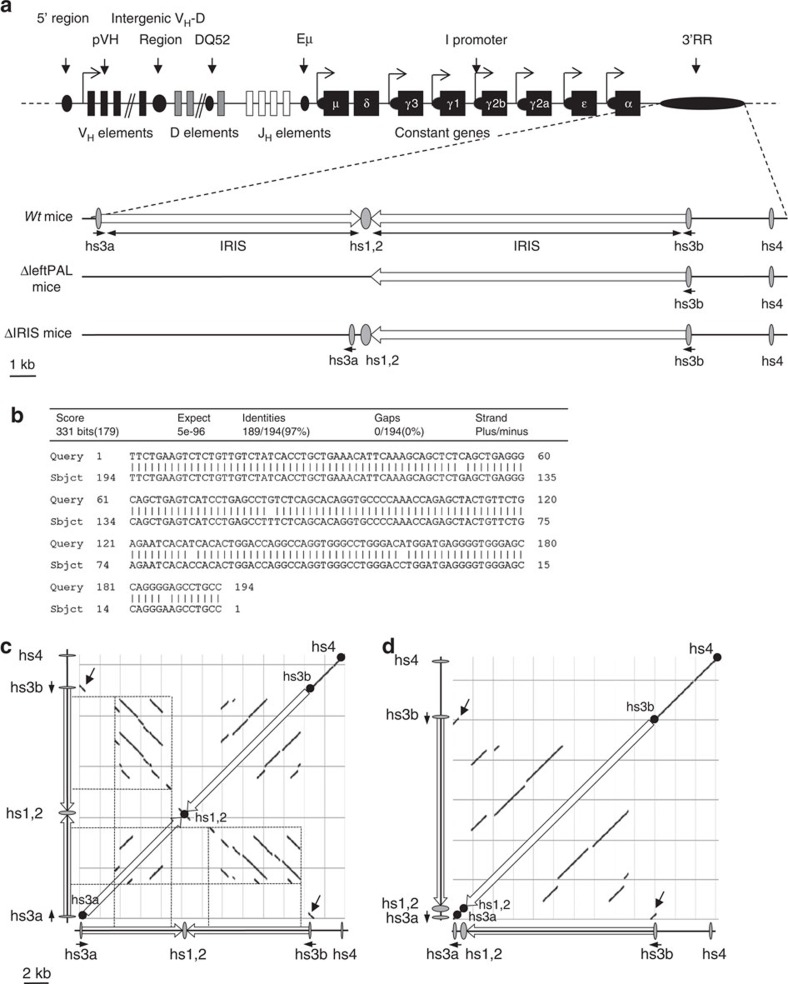
Palindromic structure of the *IgH 3'RR*. (**a**) Upper part: The mouse *IgH* locus (not on the scale). Lower part: The *3'RR* with its four enhancer elements and the *IRIS* (on the scale). ΔleftPAF and ΔIRIS mice are represented. (**b**) Sequence and homology between *hs3a* and *hs3b* enhancers (in opposite orientation in the chromosome). (**c**,**d**) DNA sequence dot-plot of the *3'RR* in *wt* (**c**) and Δ*IRIS* mice (**d**) showing self similarity. The main diagonal represents the sequence alignment with itself. Parallel lines to the main diagonal represent repetitive patterns within the sequence (that is, tandem repeat), whereas perpendicular lines to the main diagonal represent similar but inverted sequences, thus allowing to identify the palindromic structures (dotted lines). The inversion of *hs3a* enhancer (black arrows) and the deletion of the intervening sequences between *hs3a* and *hs1,2* in Δ*IRIS* mice totally disrupts the palindromic structure, despite the presence of all enhancer elements.

The location of the *3'RR* on the *IgH* locus is reported in Fig. 1a. Figure 1b reports the 97% homology between *hs3a* and *hs3b* (in inverse orientation on the chromosome). Dot-plot analysis of the *3'RR* DNA fragment encompassing *hs3a* to *hs4* reveals locations of tandem repeats and inverted sequences defining the *3'RR* palindromic structure (Fig. 1c). Inversion of *hs3a* and deletion of intervening sequences between *hs3a* and *hs1,2* in Δ*IRIS* mice totally disrupt the palindromic structure, while maintaining the presence of all *3'RR* enhancers. The ES14 cell line was used to generate ΔleftPAL and Δ*IRIS* mice. The gene-targeting vector replaced the 11.5-kb genomic fragment encompassing the *IRIS* and *hs3a*/*hs1,2* enhancers with a floxed *neo*^*R*^ cassette (ΔleftPAL mutation; Supplementary Fig. 1). Specific 5' and 3' PCR allowed the selection of 8 out of 984 clones. Another gene-targeting vector replaced the genomic fragment encompassing the *IRIS* and *hs3a*/*hs1,2* enhancers with a cassette including an inverted copy of *hs3a* plus *hs1,2* enhancer and a floxed *neo*^*R*^ cassette (Δ*IRIS* mutation; Supplementary Fig. 1). Inserting *hs3a* in inverted orientation allowed us to completely suppress any dyad symmetry around *hs1,2* without deleting any enhancer sequence (Fig. 1c,d). Specific 5' and 3' PCR allowed the selection of 6 out of 536 clones. After germline transmission, breeding with cre-expressing mice allowed the derivation of ΔleftPAL and Δ*IRIS* mice after cre-deletion of *neo*^*R*^ (Supplementary Fig. 1).

### The palindromic arrangement of *3'RR* enhancers influences SHM

**Figure 2:**
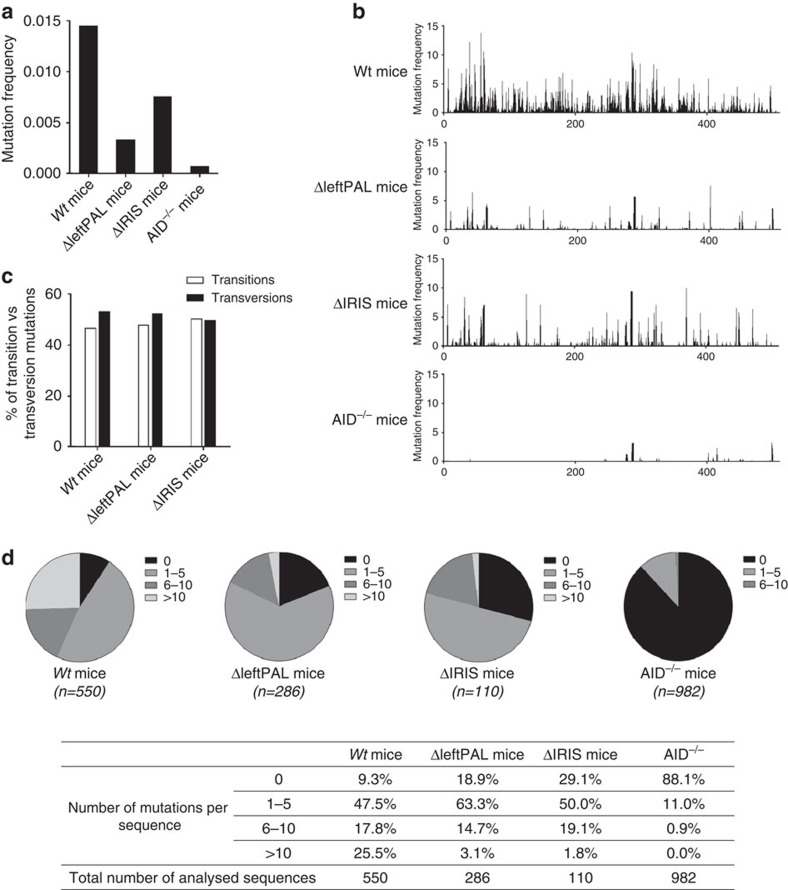
Influence of the *3'RR* palindrome on SHM. (**a**) SHM in *V*_*H*_ regions of ΔleftPAL, Δ*IRIS* and *wt* mice. Mice were immunised orally with sheep red blood cells for 2 weeks and intraperitoneally with 10 μg of LPS for 3 days. B220^+^GL7^+^Fas^+^ cells from Peyer's patches were sorted and pooled, and extracted DNA was amplified by PCR and submitted to high-throughput sequencing to evaluate SHM. *V*_*H*_ SHM values were normalised to *Jκ5* SHM values. Mean values from six mice for all genotypes were reported (8–12 weeks old, male and female). (**b**) SHM were found all along the analysed 3'*J*_*H4*_ DNA segment and hot spot of mutations were evidenced in both genotypes. Same mice as in **a**. (**c**) The proportion of transitions and transversions did not significantly differ between *wt*, ΔleftPAL and Δ*IRIS* mice. Same mice as in **a**. (**d**) Percentages of sequences with 0, 1–5, 6–10 and >10 mutations. (n): number of analysed sequences. Same mice as in **a**.

Interactions with cognate antigens recruit activated B cells into germinal centres where they undergo SHM in *V(D)J* exons for the generation of high-affinity antibodies. SHM is strongly altered in the *IgH* locus of *3'RR*-deficient mice, whereas SHM in light-chain loci remains unaltered^9^. We explored ΔleftPAL and Δ*IRIS* mice for potential SHM defects. Mice were daily immunised orally with sheep red blood cells for 2 weeks and intraperitoneally with 10 μg of LPS for 3 days. This immunisation protocol was found to be most efficient to regularly obtain *in vivo*-activated B cells in Peyer's patches^8^. Mature germinal centre B cells have a B220^+^Fas^+^GL7^+^ phenotype during normal immune responses to T-dependent antigens^16^. B220^+^GL7^+^Fas^+^ cells were sorted from immunised wild-type (*wt*), ΔleftPAL and Δ*IRIS* mice. Extracted DNA was amplified by PCR and submitted to high-throughput sequencing to evaluate SHM. As SHM in light chains is not under the *3'RR* control^9^, *IgH* SHM values along rearranged *J*_*H4*_ sequences were normalised to *Jκ5* SHM values. Mutation frequencies of 1.45% and 0.07% were found in *wt* and AID^−/−^ mice, respectively. SHM frequency was markedly reduced (by more than fourfold, at 0.33%) in ΔleftPAL mice (Fig. 2a,b). The presence of *hs3a* and *hs1,2* enhancers in Δ*IRIS* mice maintained SHM frequency at 0.75%, that is, at an intermediate level higher than ΔleftPAL mice (Fig. 2a,b) but markedly lower (by about twofold) than in *wt* mice (Fig. 2a,b). Mutations were found all along the analysed 3'*J*_*H4*_ DNA segment, but hotspots of mutations were evidenced in both genotypes. The proportion of transitions and transversions did not significantly differ between *wt*, ΔleftPAL and Δ*IRIS* mice (Fig. 2c). An increased percentage of non-mutated sequences was found in ΔleftPAL (18.9%) and Δ*IRIS* mice (29.1%) compared with *wt* mice (9.3%; Fig. 2d). When comparing the frequency of mutations per sequence, a much lower frequency of sequences carrying multiple mutations (>10 mutations) was found in ΔleftPAL (3.1%) and Δ*IRIS* (1.8%) mice compared with *wt* mice (25.5%; Fig. 2d). Thus, the palindromic structure of the *3'RR* is instrumental for SHM of rearranged *V(D)J* regions, and maintaining *hs3a* and *hs1,2* enhancers in Δ*IRIS* mice is not sufficient to preserve SHM at the *wt* level.

### The *3'RR* palindrome influences AID recruitment

**Figure 3:**
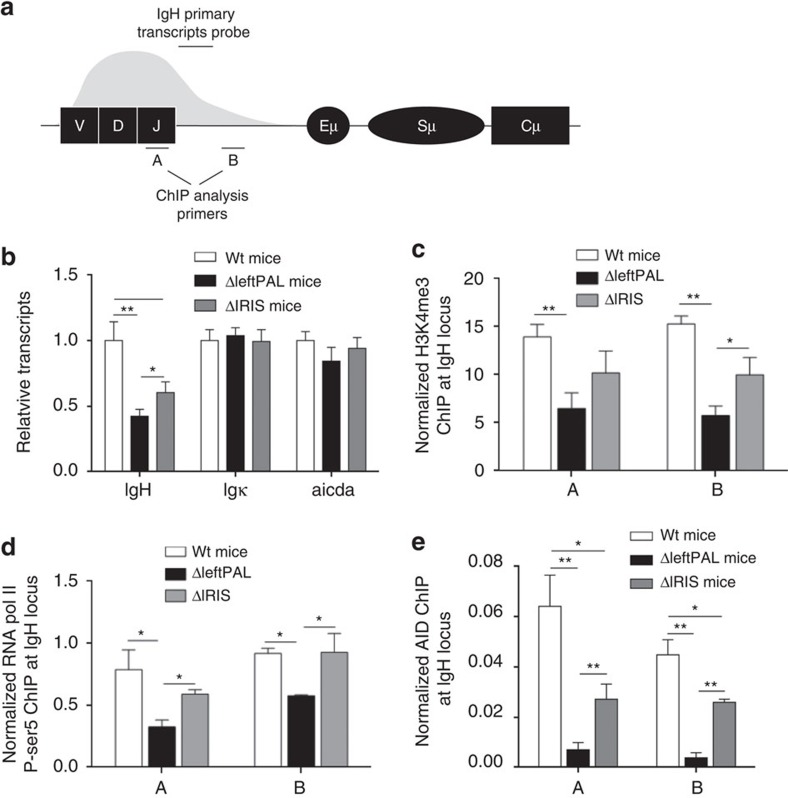
Mechanism underlying the *3'RR* palindromic effect on SHM. (**a**) Locations of probes (A,B) for ChIP experiments and PCR primers for *IgH* transcription. (**b**): *IgH*, *Igκ* and *AICDA* transcription in ΔleftPAL, Δ*IRIS* and *wt* mice. Mice were immunised orally with sheep red blood cells for 2 weeks and intraperitoneally with 10 μg of LPS for 3 days. Peyer's patch cell RNA was extracted and transcripts were amplified by real-time PCR. Data are the mean±s.e.m. of six independent experiments with one mouse per genotype (8–12 weeks old, male and female). *P<0.05 and **P<0.001 (Mann–Whitney's *U*-test for significance). Values were normalised to *GAPDH* transcripts. (**c**) ChIP analysis of H3K4me3 in *V*_*H*_ regions in Peyer's patch cells in ΔleftPAL, Δ*IRIS* and *wt* mice. Background ChIP signals from mock samples with irrelevant antibody were subtracted. ChIP values were normalised to the total input DNA. Data are the mean±s.e.m. of four independent experiments (8- to 12-week-old mice, male and female). *P<0.05, **P<0.01 (Mann–Whitney *U*-test). ChIP experiments were done in A and B locations (as in **a**). Same immunisation protocol as in **b**. (**d**) ChIP analysis of pol II paused in *V*_*H*_ regions in Peyer's patches cells in ΔleftPAL, Δ*IRIS* and *wt* mice. Data are the mean±s.e.m. of four independent experiments (8- to 12-week-old mice, male and female). *P<0.05, **P<0.01 (Mann–Whitney *U*-test). Same immunisation protocol as in **b**. (**e**) ChIP analysis of AID recruitment in *V*_*H*_ regions in Peyer's patch cells in ΔleftPAL, Δ*IRIS* and *wt* mice. Data are the mean±s.e.m. of five independent experiments (8- to 12-week-old mice, male and female). *P<0.05, **P<0.01 (Mann–Whitney *U*-test). Same immunisation protocol as in **b**.

We next investigated the mechanism underlying the SHM alteration. SHM correlates with transcription^17^. The partial *IgH* transcription defect observed in *hs3b*/*hs4*-deficient mouse resting B cells did not lead to any significant SHM decrease in germinal centre B cells^18^. The situation is quite different in *3'RR*-deficient mice, in which a partial *IgH* transcription defect was again observed, but then it was associated with a nearly complete *V(D)J* SHM blockade in activated germinal centre B cells^9^. In the present study, *IgH* primary transcription (location of the probe is shown in Fig. 3a) is maintained at fairly high levels in germinal centre B cells of ΔleftPAL mice and even slightly higher in Δ*IRIS* mice, thus clearly uncoupling the SHM defect from any major *V(D)J* transcription defect (Fig. 3b). As positive controls, no alteration was found for *Igκ* transcripts (nor for *AID* transcripts) in ΔleftPAL and Δ*IRIS* mice (Fig. 3b). Together with transcription, modified histones, characteristic of active chromatin, constitute hallmarks of the accessibility to SHM factors. ChIP experiments (probes located are shown in Fig. 3a) indicated that trimethylation of lysine 4 in histone H3 (H3K4me3) was markedly decreased in activated Peyer's patch cells of ΔleftPAL mice but partially preserved in Δ*IRIS* mice (Fig. 3c). This shows a synergistic role of enhancers and of the palindromic architecture for induction of epigenetic modifications and chromatin accessibility. Stalling of RNA pol II onto the *IgH V* region is required for AID recruitment during SHM in germinal centre B cells. As shown in Fig. 3d, paused RNA pol II loading was markedly reduced in activated Peyer's patch cells of ΔleftPAL mice and almost normal in Δ*IRIS* mice. In contrast, AID recruitment is strongly affected in both ΔleftPAL and Δ*IRIS* mice compared with *wt* mice (Fig. 3e). Our data thus suggest that the *3'RR* palindrome strongly contributes to the efficient recruitment of AID onto the *V(D)J* region during SHM.

### The *3'RR* palindrome weakly influences germline transcription

**Figure 4:**
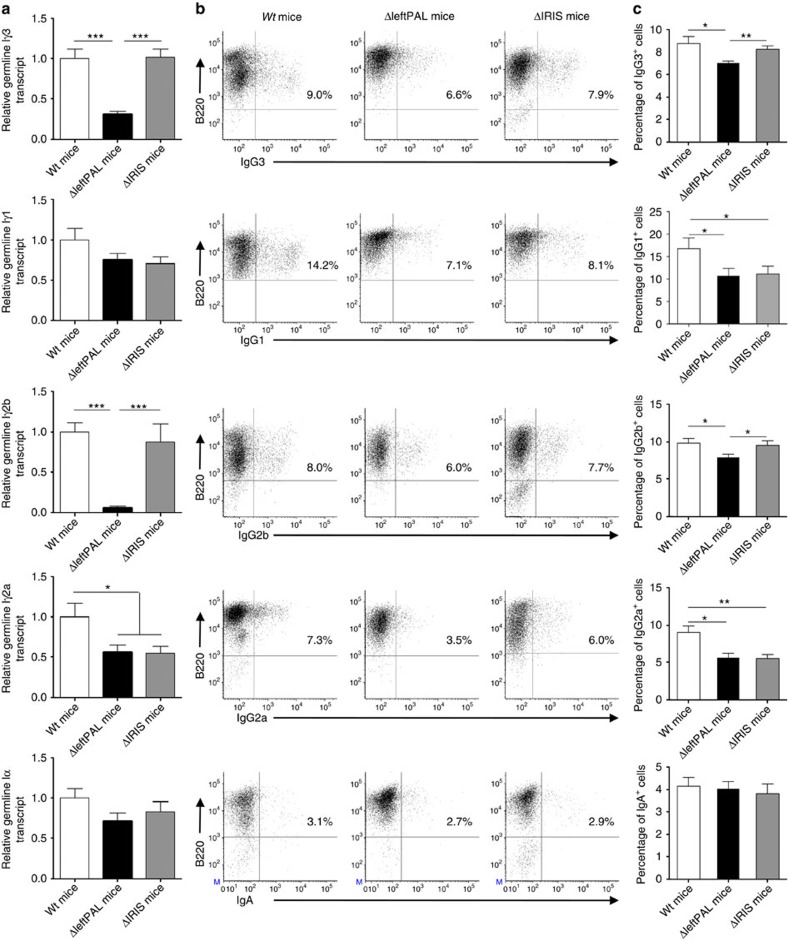
Influence of the *3'RR* palindrome on GLT and CSR. (**a**) GLT in B splenocytes of ΔleftPAL, Δ*IRIS* and *wt* mice. Cells were stimulated with LPS±IL-4, INFγ and TGFβ for 2 days. *Iγ*_*1*_*-C*_*γ1*_, *Iγ*_*2a*_*-C*_*γ2a*_, *Iγ*_*2b*_*-C*_*γ2b*_, *Iγ*_*3*_*-C*_*γ3*_ and *I*_*α*_*-C*_*α*_ GL transcription was investigated by real-time PCR. Mean±s.e.m. of six independent experiments with one mouse (8–12 weeks old, male and female). *P<0.05 and ***P<0.0001 (Mann–Whitney *U*-test for significance). Values were normalised to *GAPDH* transcripts. (**b**) CSR in B splenocytes of ΔleftPAL, Δ*IRIS* and *wt* mice. Cells were stimulated with LPS±IL-4, INFγ and TGFβ for 3 days. Cells were then labelled with anti-B220-APC antibodies and anti-IgG_1_-, anti-IgG_2a_-, anti-IgG_2b_-, anti-IgG_3_- and anti-IgA-FITC antibodies. One representative experiment out of six (one mouse per experiment) is shown (8- to 12-week-old mice, male and female). (**c**) Mean±s.e.m. of six independent experiments of CSR with one mouse (8–12 weeks old, male and female). *P<0.05 and **P<0.001 (Mann–Whitney *U*-test for significance).

Germline transcription (GLT) of *C*_*H*_ gene is a known prerequisite of CSR. We evaluated GLT using real-time PCR on LPS only (*I*_*γ3*_*-C*_*γ3*_*, I*_*γ2b*_*-C*_*γ2b*_), LPS plus IL4- (*I*_*γ1*_*-C*_*γ1*_), LPS plus TGFβ- (*I*_*α*_*-C*_*α*_) and LPS plus INFγ- (*I*_*γ2a*_*-C*_*γ2a*_) activated splenic B cells from *wt*, ΔleftPAL and Δ*IRIS* mice. As shown in Fig. 4a, GLT was heterogeneously affected in ΔleftPAL mice, more or less for *I*_*γ3*_*-C*_*γ3*_, *I*_*γ2a*_*-C*_*γ2a*_ and *I*_*γ2b*_*-C*_*γ2b*_, whereas *I*_*α*_*-C*_*α*_ and *I*_*γ1*_*-C*_*γ1*_ were preserved. Alterations were milder and restricted to *I*_*γ2a*_*-C*_*γ2a*_ GLT in Δ*IRIS* mice (Fig. 4a). These data identify GLT as a basic activity of *3'RR* transcriptional enhancers that can arise almost independently from their inclusion into the *3'RR* palindrome. Accordingly, combined deletion of *hs3b*+*hs4* elements, although minimally truncating the *3'RR* palindrome, had a marked role in GLT^19^. It also has to be acknowledged that the various *C* genes are not equally affected by *3'RR* defects and that *C*_*γ1*_ GLT was only affected by the whole *3'RR* deletion^6^,^20^, whereas *C*_*α*_ GLT is sensitive to deletions encompassing the *hs4* enhancer element^6^,^21^.

### The *3'RR* palindrome weakly influences CSR

To determine whether results on GLT translated to a decreased CSR, we appreciated by flow cytometry the number of cells switching to a particular isotype after *in vitro* stimulation. Flow cytometric analysis allowed the counting of cells and the study of surface expression of class-switched isotypes on LPS only, LPS plus IL4-, LPS plus TGFβ- and LPS plus INFγ-activated splenic B cells from *wt* and ΔleftPAL mice. A pattern almost mirroring the results of GLT (except for γ1) was found (Fig. 4b,c). Lowered CSR was found for IgG_3_, IgG_2a_ and IgG_2b_, but not IgA. CSR towards IgG_1_ was slightly reduced despite unchanged GLT. Maintaining the presence of all enhancer elements in Δ*IRIS* mice preserved CSR towards IgG_3_ and IgG_2b_ but not IgG_2a_ and IgG_1_. IgA CSR was in parallel normal, similar to ΔleftPAL mice. Downstream of GLT, we observed efficient preservation of the *in vitro* IgG_3_ and IgG_2b_ CSR when enhancers were maintained. Our data confirm the particular status of γ_1_ and α isotypes with respect to the *3'RR*-dependent regulation of CSR control. IgA CSR is only sensitive to deletion encompassing the *hs4* enhancer element^6^,^20^, and recently an enhancer-RNA-expressing element called Inc-RNA-CSR was reported to promote CSR towards α by stimulating activity of the *3'RR* via a long-distance interaction with the *hs4* region^21^.

### The *3'RR* palindrome and chromatin accessibility

**Figure 5:**
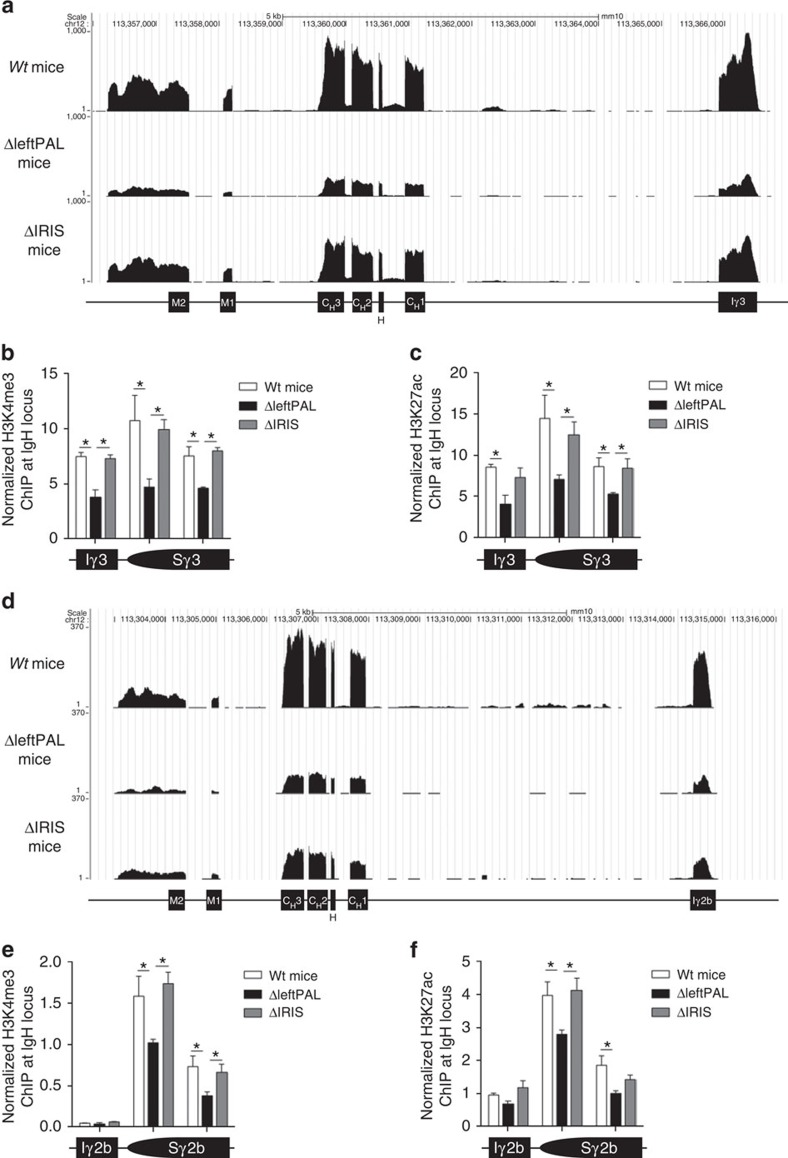
Influence of the *3'RR* palindrome on *IgH* transcription and activation of epigenetic marks during CSR. (**a**) *Iγ*_*3*_*-C*_*γ3*_ transcription in ΔleftPAL, Δ*IRIS* and *wt* mice. CD43-depleted splenocytes were cultured for 2 days with 5 μg of LPS. RNAseq experiments were performed after depletion of rRNA. Data are the mean of two independent experiments with three mice per genotype (8- to 12-week-old-male mice) (**b**) ChIP analysis of H3K4me3 in *I*γ_*3*_*-C*_*γ3*_ in ΔleftPAL, Δ*IRIS* and *wt* mice. Data are the mean±s.e.m. of four independent experiments (8- to 12-week-old mice, male and female). *P<0.05 (Mann–Whitney *U*-test). (**c**) ChIP analysis of H3K27ac in *Iγ*_*3*_*-C*_*γ3*_ in ΔleftPAL, Δ*IRIS* and *wt* mice. Data are the mean±s.e.m. of four independent experiments (8- to 12-week-old mice, male and female). *P<0.05 (Mann–Whitney *U*-test). (**d**) *Iγ*_*2b*_*-C*_*γ2b*_ transcription (RNAseq experiments) in ΔleftPAL, Δ*IRIS* and *wt* mice. Same mice as in **a**. (**e**) ChIP analysis of H3K4me3 in *Iγ*_*2b*_*-C*_*γ2b*_ in ΔleftPAL, Δ*IRIS* and *wt* mice. Data are the mean±s.e.m. of four independent experiments (8- to 12-week-old mice, male and female). *P<0.05 (Mann–Whitney *U*-test). (**f**) ChIP analysis of H3K27ac in *Iγ*_*2b*_*-C*_*γ2b*_ in ΔleftPAL, Δ*IRIS* and *wt* mice. Data are the mean±s.e.m. of four independent experiments (8- to 12-week-old mice, male and female). *P<0.05 (Mann–Whitney *U*-test).

Molecular analysis of CSR has shown that the *3'RR* notably promotes CSR by acting on its initial steps (GLT and histone modifications)^20^. Together with DNA transcription, several epigenetic marks involved in the targeting of the CSR machinery to *S* regions primed for CSR constitute hallmarks of CSR accessibility^22^,^23^. RNAseq experiments showed that *C*_*γ3*_ and *C*_*γ2b*_ transcription (Fig. 5a and d, respectively) was markedly reduced in ΔleftPAL mice compared with *wt* mice. The presence of *hs3a* and *hs1,2* enhancers in Δ*IRIS* mice preserved *C*_*γ3*_ and *C*_*γ2b*_ transcription. Levels of H3K4me3 (Fig. 5b,e) and H3K27ac (Fig. 5c,f) in the *I*_*γ3*_-*S*_*γ3*_-*C*_*γ3*_ and *I*_γ2b_*-S*_γ2b_*-C*_γ2b_ regions (during IgG_3_ and IgG_2b_ CSR, respectively) were reduced only on deletion of *3'RR* enhancers but preserved in Δ*IRIS* mice. This suggests that *3'RR* effects on CSR mostly rely on the presence of all four *3'RR* enhancers, more or less independently from their rigorous palindromic arrangement.

### The *3'RR* palindrome and B-cell fate

**Figure 6:**
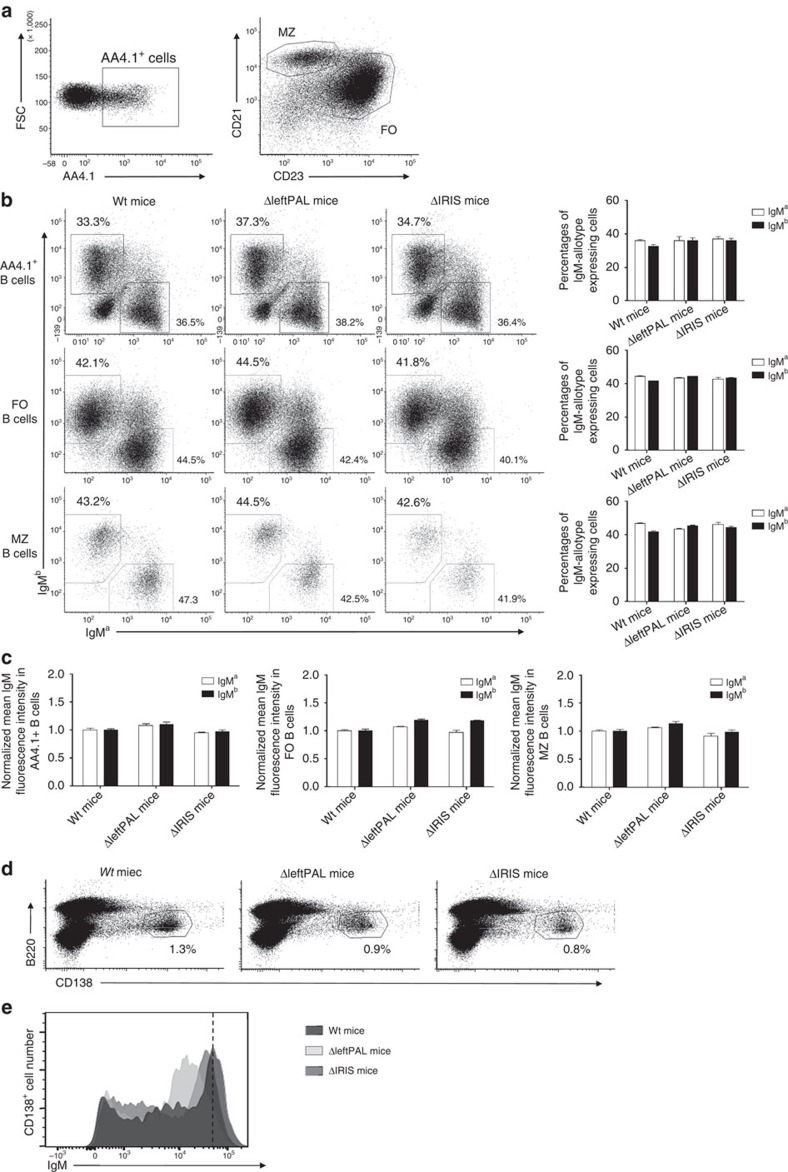
Influence of the *3'RR* palindrome on BCR expression and B-cell fate. (**a**) Flow cytometry analysis of transitional (TR) B cells (AA4.1^+^), follicular (FO) B cells (CD21^low^CD23^high^) and marginal zone (MZ) B cells (CD21^high^CD23^low^). One representative experiment out of five is shown (8- to 12-week-old mice, male and female). (**b**) TR, FO and MZ B cells expressing the *a* or *b* allele in a^ΔleftPAL^/b^*wt*^, a^ΔIRIS^ /b^*wt*^ and a^*wt*^/b^*wt*^ mice. One representative experiment out of five is shown (left part). Mean±s.e.m. of five mice for all genotypes (right part; 8–12-week-old mice, male and female). (**c**) Mean±s.e.m. of membrane IgM densities on TR, FO and MZ B cells expressing the *a* or *b* allele in a^ΔleftPAL^/b^*wt*^, a^ΔIRIS^ /b^*wt*^ and a^*wt*^/b^*wt*^ mice (8–12-week-old mice, male and female). (**d**) Flow cytometry analysis of plasmablasts (B220^+^CD138^+^) in spleen of homozygous a^ΔleftPAL^/b^*wt*^, a^ΔIRIS^ /b^*wt*^ and a^*wt*^/b^*wt*^ mice. One representative experiment out of three is shown (8- to 12-week-old mice, male and female). (**e**) Expression of IgM on plasmablasts of homozygous ΔleftPAL, ΔIRIS and *wt* mice. One representative experiment out of three is shown (8- to 12-week-old mice, male and female).

The full deletion of the *3'RR* was reported to affect membrane IgM density and to modulate the B-cell fate towards less marginal zone B cells^5^. We generated heterozygous *IgH* a^ΔleftPAL^/b^*wt*^ mice and *IgH* a^Δ*IRIS*^/b^*wt*^ mice by crossing homozygous ΔleftPAL mice (*IgH* a^ΔleftPAL^/a^ΔleftPAL^) and ΔIRIS mice (*IgH* a^Δ*IRIS*^/a^Δ*IRIS*^) with C57BL/6 mice (*IgH* b^*wt*^/b^*wt*^). Analysis of splenic B cells with IgM-allotype-specific antibodies indicated similar percentages of transitional B cells (B220^+^AA4.1^+^), follicular B cells (B220^+^CD21^low^CD23^high^) and marginal zone B cells (B220^+^CD21^high^CD23^low^) expressing either the *a* or *b* allotype in a^ΔleftPAL^/b^*wt*^ and *IgH* a^Δ*IRIS*^/b^*wt*^ mice (Fig. 6a,b) and no defect in membrane IgM density (Fig. 6c). Percentages of B220^+^CD138^+^ plasmablasts were not different in ΔleftPAL, Δ*IRIS* and *wt* mice (Fig. 6d). The lowered IgM density observed in CD138^+^ plasmablasts is almost restored in Δ*IRIS* mice (Fig. 6e). This again suggests that most of the effects of *3'RR* enhancers on IgM expression and relevant B-cell fate are simply provided by the presence of all four *3'RR* enhancers, without requiring their rigorous palindrome arrangement.

### The *3'RR* palindrome and Ig synthesis

**Figure 7:**
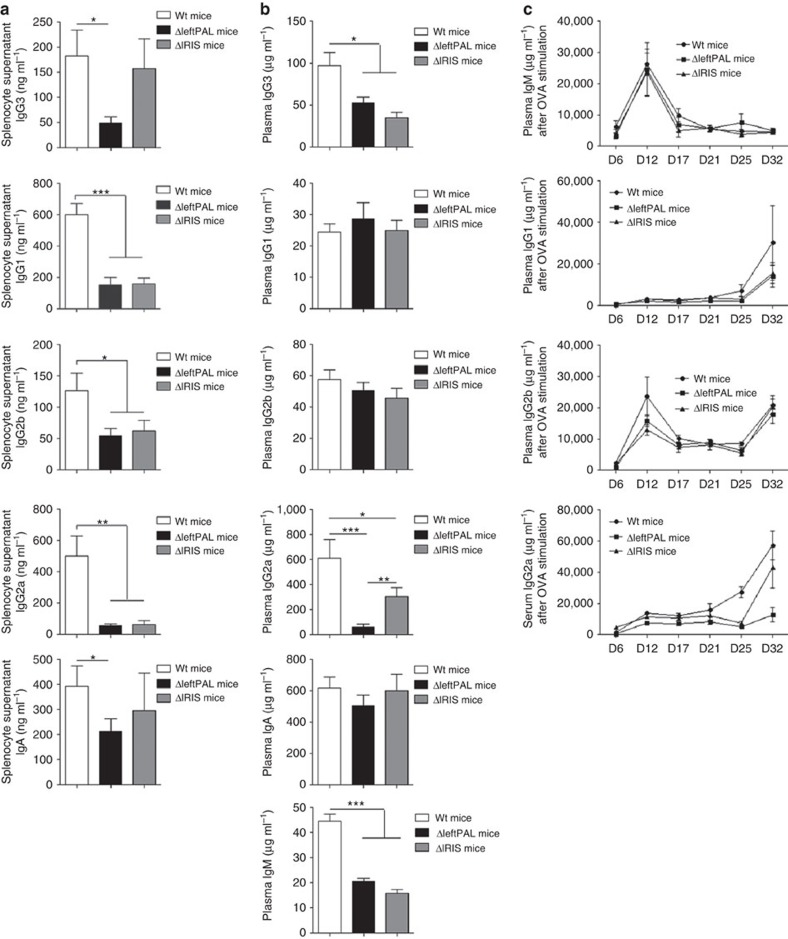
Influence of the *3'RR* palindrome on Ig synthesis. (**a**) ELISA analysis of IgG_1_, IgG_2a_, IgG_2b_, IgG_3_ and IgA in supernatants of LPS±IL-4-, INFγ- and TGFβ-stimulated splenocytes of ΔleftPAL, Δ*IRIS* and *wt* mice. Data are the mean±s.e.m. of eight experiments with one mouse (8–12 weeks old, male and female). *P<0.05, **P<0.01 and **P<0.001 (Mann–Whitney *U*-test for significance). (**b**) ELISA analysis of IgM, IgG_1_, IgG_2a_, IgG_2b_, IgG_3_ and IgA in plasma of 10 ΔleftPAL, 9 Δ*IRIS* mice and 18 *wt* mice (8 weeks old, male and female). Mean±s.e.m. *P<0.05, **P<0.01 and **P<0.001 (Mann–Whitney *U*-test for significance). (**c**) Ovalbumin-specific IgM, IgG_1_, IgG_2a_ and IgG_2b_. Antibody levels, detected by ELISA, are expressed in arbitrary units by comparison with control plasma values. Time after immunisation is indicated in days. Each point is the mean±s.e.m. of plasma determinations from six mice for each genotype (8–12 weeks old, male and female). One representative experiment out of two is shown.

Ig production was assessed *in vitro* through stimulation of splenocytes with LPS and/or cytokines. It was significantly affected in both mutant strains of mice, although this phenotype was aggravated in ΔleftPAL mice lacking two enhancers. IgG_2b_ (LPS stimulation), IgG_1_ (LPS+IL4 stimulation), IgG_2a_ (LPS+INFγ stimulation) and IgA (LPS+TGFβ stimulation) syntheses were markedly reduced in ΔleftPAL compared with *wt* mice (Fig. 7a). Similar alterations were observed in Δ*IRIS* mice, except that they were eventually less pronounced and spared IgG_3_ and IgA secretion (Fig. 7a). We next investigated Ig production and plasma accumulation *in vivo* and again found similar alterations in both mutant strains, which mostly showed defects for IgM, IgG_3_ and IgG_2a_ plasma levels, whereas IgG_1_, IgG_2b_ and IgA were preserved (Fig. 7b). Although statistically significant in both cases, the plasma IgG_3_ and IgG_2a_ levels were less markedly decreased in Δ*IRIS* mice than in mice with the dual deletion of *hs3b* and *hs4* enhancers^19^. Although these total Ig-level defects were clear, evaluation of specific circulating antibody levels after ovalbumin immunisation did not show major alteration, neither in ΔleftPAL nor in Δ*IRIS* mice, suggesting that the intrinsic B-cell defect was compensated on specific antigenic challenge (with potentially a trend of lower levels of IgG1 and IgG2a antibodies in mutant animals; Fig. 7c).

## Discussion

**Figure 8:**
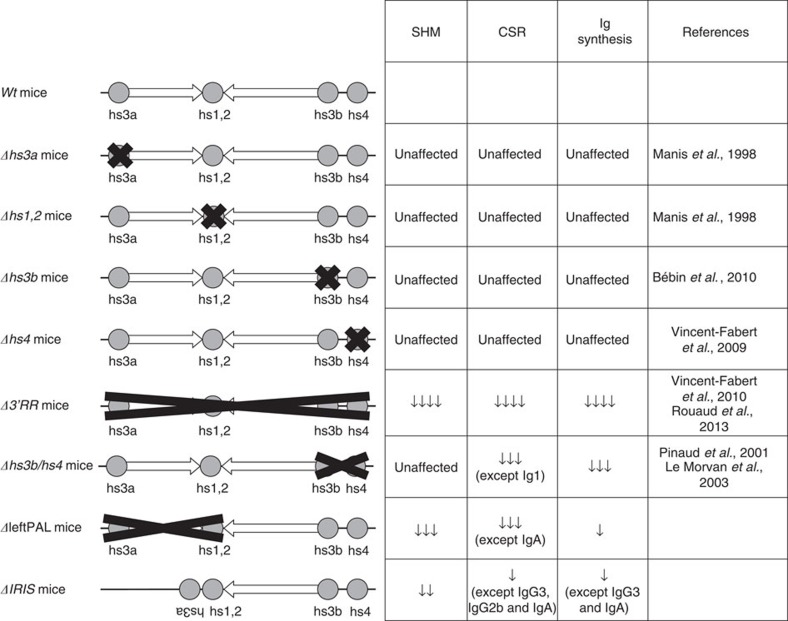
Mutants of the *3'RR* enhancers. References and main results are reported and compared with ΔleftPAL and Δ*IRIS* mice.

The *IgH 3'RR* is a large and complex *cis*-regulatory region with a unique and striking palindromic architecture (Fig. 1) for which a potential functional role has been long questioned. Because the previously reported focal genomic alterations of the *3'RR* could not address the issue of a role for the *3'RR* architecture^6^,^19^,^24^,^25^,^26^,^27^ (Fig. 8), we generated two different mice with complete disruption of the palindromic architecture, either by globally deleting the first-half part of the *3'RR* (including *hs3a* and *hs1,2* enhancers) or by removing all inverted repeats while preserving all enhancers (but with *hs3* enhancer disposed as direct repeats) and compared them. SHM in *V*_*H*_ genes was markedly affected by the palindromic deconstruction. Keeping *hs3a* and *hs1,2* enhancer intact in Δ*IRIS* mice prevented the fall in histone modification and RNA pol II pausing but nevertheless affected AID recruitment in *V*_*H*_ genes. Taken together, this indicates that, even when all enhancer elements were present, breaking the palindrome symmetry by deleting half of inter-enhancer intervening sequences plus flipping *hs3a* in a direct repeat orientation affected SHM at a level similar to ΔleftPAL mice also lacking *hs3a* and *hs1,2*. Although a role for the non-conserved intervening sequences by themselves cannot be excluded, these data strongly suggest a role for the palindromic *3'RR* architecture during SHM. Indeed, although the absence of any inter-species sequence identity between *3'RR IRIS* suggests that they do not include major functional motif, conservation of their dyad symmetry architecture around *hs1,2* by contrast suggests an evolutionary pressure on the palindromic structure itself^15^. Our data fully support the idea that this pressure on the *3'RR* architecture connects with a functional importance for germinal centre B-cell response and thus for optimal humoral immunity. On the basis of previously published data, one can also suggest that the *IgH 3'RR* is somehow split into functional modules. The 25-kb-long palindrome seems to have a strong role in SHM, whereas *hs4* deletion alone only affected membrane IgM expression in resting B cells^27^, and the *hs3b*+*hs4* deletion affected IgM expression and CSR but not SHM^18^,^19^. These data do not formally exclude the hypothesis that the reduction in distances between the enhancers alone accounts for our SHM observation. For other enhancers, dependence on proper spatial organisation and spacing has been reported^28^. However, we think that data regarding the *3'RR* are much more in favour of an architectural role of the repeats, contributing to a given functional secondary or tertiary structure of either the *3'RR* and/or the whole *IgH* locus. A contribution of transcription to *3'RR* conformation is also suggested by our previous characterisation of *3'RR* eRNA^8^, and the recent demonstration that long non-coding RNA coming from distal IncRNA-CSR element positively regulates the activity of the *3'RR* (ref. 21).

Combined effect of the four *3'RR* enhancers was not sufficient alone for an efficient AID recruitment and thus for a normal SHM rate. Interestingly, a previously reported *3'RR* deletion sparing most of its palindromic part but removing its two last enhancers (*hs3b*+*hs4*) had no effect on SHM^18^, but it had major effects on CSR^9^, whereas complete deletion of the *3'RR* was shown to simultaneously affect CSR and SHM^6^,^9^. In the present study, analysis of ΔleftPAL and Δ*IRIS* mice with a deconstruction of the *3'RR* palindrome only had a minor effect on CSR with reduced CSR towards γ_3_ and γ_2b_ connected with decreased γ_3_ and γ_2b_ GLT. The *3'RR*-induced CSR control is complex, as CSR towards α is mostly dependent on the *hs4* enhancer^21^, and as CSR to γ_1_ appears as at least partially *3'RR*-independent^6^,^20^. These differences probably relate to the specific structures of germline promoters and of *S* regions, as the number of G-clusters to initiate R loops, the number of WGCW sites for AID deamination and distance to promoter are of key importance for CSR efficiency^29^. Thereby, *S*_*α*_ has been demonstrated to be able to form R-loops more readily than other isotypes, thus allowing AID to induce the double-strand breaks (DSBs) required to initiate CSR, because of the short distance between *I*_*α*_ and *C*_*α*_ (ref. 30). Transcription is suggested to play a key role both in SHM and CSR. However, the induction of these two processes is different. Although CSR relies mostly on transcription and the repetitive sequences of *S* regions to produce ssDNA and recruit AID, SHM requires the presence of many co-factors for efficient AID targeting and, thus, may be more dependent on the 3D structure of the *3'RR*, which may allow a precise regulation of this mechanism. Moreover, CSR is a ‘yes' or ‘no' process, which might still be able to occur even when the frequency of AID lesions within downstream *S* region is decreased (especially in conditions with a *3'RR* deletion, where double-strand breaks still occur at high rate in the upstream *S*_*μ*_ region^20^. In addition, CSR and SHM require the activity of different parts of the AID molecule and different partners of AID, such as 14-3-3 and KAP1/HP1 in the case of CSR^31^. Finally, it has been demonstrated that *V* and *S* regions use different mechanisms to expose ssDNA to AID. Although *S* regions form long R-loops because of to the high level of transcription and their repetitive sequence, *V* regions display short patches of ssDNA that require the assembly of protein–DNA complex^32^. AID is, evolutionarily, the first enzyme known to improve immune diversity by SHM, and it is present as early as the primordial jawed vertebrates; AID-induced CSR begins later, with the first amphibians^32^,^33^,^34^.

In conclusion, SHM requires both *3'RR* enhancers and its palindromic architecture, whereas CSR relies mostly on the enhancers, which are sufficient for GLT and accessibility of the locus. Inverted repeats may function by positioning the *3'RR* in its optimal 3D configuration, together with eRNA, IncRNA and transcription factor in order to stabilise the chromosomal loops that most efficiently recruit AID downstream of *pV*_*H*_ promoters^35^. It is of interest to note that CSR and the increased *IgH* transcription occurring at the plasma cell stages do not require these elements, reinforcing the concept that CSR and SHM are mechanistically differently controlled by the *3'RR*. Understanding differences of the pathways that contribute to CSR and SHM will help us understand the mechanisms for antibody regulation and diversity. Our present results indicate that the *3'RR* regulates AID-induced SHM and CSR by different mechanisms, underline the complexity of action of this major *cis*-regulatory element and demonstrate that not only enhancers included in the *3'RR* but also the global palindromic architecture of this region are critical determinants of its function.

## Methods

### Mice

In all, 129 *wt* mice and C57Bl/6 *wt* mice (from Charles Rivers Laboratories, France), as well as ΔLeftPAL and Δ*IRIS* mice (from UMR CNRS 7276, Limoges, France; in a 129 background), were used. Our research has been approved by our local ethics committee review board (Comité Régional d'Ethique sur l'Expérimentation Animale du Limousin, Limoges, France) and has been carried out according to the European guidelines for animal experimentation.

### Vector construction and embryonic stem cell screening

A neomycin-resistance gene (*neo*^R^) flanked by *loxP* sites was stuck in between 5' and 3' arms. At the 5' end, a phosphoglucokinase promoter-herpes simplex virus thymidine kinase gene (*Tk*) was included to permit negative selection against random integration. Cells of the embryonic stem (ES) cell line E14 were transfected with linearised vector by electroporation and selected using 300 μg ml^−1^ geneticin and 2 μg ml^−1^ gancyclovir. The ES cell line E14 was derived from the inbred mouse strain 129. PCR analysis with primers 5' and 3' of the construct identified recombinants. ES clones showing homologous recombination were injected into C57Bl/6 blastocysts, and the resulting chimeras were mated with C57Bl/6 animals. Germline transmission in heterozygous mutant mice was checked by specific PCR. Mutant mice were mated with cre-transgenic mice. The progeny was checked by PCR for the occurrence of a cre-mediated deletion of the *neo*^R^ gene. ΔleftPAL and Δ*IRIS* homozygous mice were checked by PCR. The various PCR primers used for screening the ΔleftPAL and Δ*IRIS* mice are reported in the Supplementary Table 1 and located in the Supplementary Fig. 1.

### Blood sampling

Blood samples were recovered from transgenic mice and *wt* controls with heparinised needles. Ten-week-old animals (male and female) were used. Plasma samples were recovered by centrifugation and stored at −20 °C until use.

### Spleen cell cultures for CSR and Ig determinations

Single-cell suspensions of CD43^−^ spleen cells of *wt*, ΔleftPAL and Δ*IRIS* mice (8–12 weeks old, male and female) were cultured for 3 days at 1 × 10^6^ cells per ml in RPMI 1640 with 10% fetal calf serum, 5 μg ml^−1^ LPS with or without 20 ng ml^−1^ IL-4, 2 ng ml^−1^ TGFβ and 2 ng ml^−1^ INFγ (PeproTech, Rocky Hill, NJ)^6^,^36^. At day 3, 1 × 10^6^ cells were cultured for 24 h in growth medium without LPS+cytokine. Supernatants were recovered and stored at −20 °C until use. At day 3, cultured splenic B cells were incubated with anti-B220-SpectralRed (PC5)-labelled antibodies (Biolegend, ref: 103212) and anti-IgG_1_- (ref: 107020), anti-IgG_2a_- (ref: 108002), anti-IgG_2b_- (ref: 109002), anti-IgG_3_- (ref: 110002) and anti-IgA- (ref: 104002) fluorescein-isothiocyanate (FITC)-labelled antibodies (Southern Biotechnologies), and then analysed on a Fortessa LSR2 (Beckton-Dickinson)^6^. All antibodies were at a concentration of 10 μg ml^−1^.

### Real-time quantitative PCR of I_x_-C_x_ GL transcription

Three-day *in vitro*-stimulated splenocytes (LPS+appropriated cytokines) were collected and RNA was extracted for investigation of *I*_*x*_*-C*_*x*_ transcripts. RNA and cDNA were prepared using standard techniques. Quantitative PCR was performed using power SYBR green (Applied Biosystems). PCR primers used for determinations of *I*_*x*_*-C*_*x*_ transcripts are reported in the Supplementary Table 1.

### Immunisation

For immunisation experiments, batches of 8-week-old mice were used (6 mice per genotype, male and female). The first immunisation was performed with 50 μg of chicken ovalbumin per animal in 50% complete Freund's adjuvant and a second immunisation was performed 13 days later with the same amount of antigen in 50% incomplete Freund adjuvant. Immunised mice were eye-bled at various intervals during the immunisation protocol, and plasma was analysed for the presence of ovalbumin-specific IgM, IgG_1_, IgG_2a_ and IgG_2b_ by ELISA^18^.

### Antibody determinations

Specific ELISAs were performed as follows^6^,^18^: ELISAs for specific IgM, IgG_1_, IgG_2a_ and IgG_2b_ were performed in polycarbonate 96-multiwell plates coated overnight at 4 °C with 100 μl of 10 mg ml^−1^ ovalbumin solution in 0.05 M Na_2_CO_3_ buffer. After washing, a blocking step was performed with gelatin (2 mg ml^−1^) in PBS buffer. After washing, 50 μl of assayed plasma or control plasma was diluted into successive wells (first dilution to 1:50) in gelatin (2 mg ml^−1^) in PBS buffer and incubated for 2 h at 37 °C. The positive control consisted of a pool of plasma from ovalbumin-immunised *wt* mice (the same control plasma was used in all ELISAs). After washing, 100 μl per well of appropriate conjugated antibodies were added and adsorbed for 2 h at 37 °C. Alkaline phosphatase (AP)-conjugated goat antisera specific for mouse IgM (Southern Biotechnologies, ref: 102104), IgG_1_ (Southern Biotechnologies, ref: 107004), IgG_2a_ (Southern Biotechnologies, ref: 1080004) and IgG_2b_ (Beckman Coulter, ref: 731943) were used at a concentration of 1 μg ml^−1^. After washing, AP activity was assayed using AP substrate, and enzymatic reactions were stopped with 3 M NaOH. The optical density was measured at 400 nm. Diluted plasmas were compared with the titration curve obtained on the same multiwell plate, which allowed the quantification of ovalbumin-specific antibodies in arbitrary units. Culture supernatants and plasma (first dilution to 1:50) from ΔleftPAL, Δ*IRIS* and *wt* mice were analysed for the presence of the various Ig classes (IgM, IgG_1_, IgG_2b_, IgG_2b_, IgG_3_ and IgA) by ELISA, as previously described above^6^,^18^,^20^, except for the coating made with suitable capture antibodies (2 μg ml^−1^ for IgM (Southern Biotechnologies, ref: 902001), IgG_1_ (Southern Biotechnologies, ref: 107001), IgG_2a_ (Southern Biotechnologies, ref: 731956) and IgG_2b_ (Southern Biotechnologies, ref: 731940), 3 μg ml^−1^ for IgG_3_ (Southern Biotechnologies, ref: 732371) and 4 μg ml^−1^ for IgA (Southern Biotechnologies, ref: 104001), blocking performed with 3% BSA in PBS, AP-conjugated goat antisera specific for mouse IgA (Southern Biotechnologies, ref: 104004) and IgG_3_ (Beckman Coulter, ref: 732374), and for standards made with specific antisera for IgM, IgG_1_, IgG_2a_, IgG_2b_, IgG_3_ and IgA (Southern Biotechnologies)).

### ChIP experiments

Mice immunisations were performed orally with sheep red blood cells for 2 weeks and intraperitoneally with 10 μg of LPS for 1 week (8–12 weeks old, male and female). ChIP experiments were done on freshly isolated Peyer's patches cells, as previously described^8^. In brief, 4 × 10^6^ B cells were cross-linked at room temperature for 15 min in 15 ml of PBS with 1% formaldehyde. The reaction was quenched with 0.125 M glycine. After lysis, chromatin was sonicated to 0.5–1 kb using a Vibracell 75043 (Thermo Fisher Scientific). After dilution in ChIP buffer (0.01% SDS, 1.1% Triton X-100, 1.2 mM EDTA, 16.7 mM Tris-HCl, pH 8.1 and 167 mM NaCl), chromatin was precleared by rotating for 2 h at 4 °C with 50 ml of 50% protein A/G slurry (0.2 m ml^−1^ sheared salmon sperm DNA, 0.5 m ml^−1^ BSA and 50% protein A/G; Sigma). In all, 0.1 × 10^6^ cell equivalents were saved as input, and 2 × 10^6^ cell equivalents were incubated overnight with anti-AID or control antibodies. Immune complexes were precipitated by the addition of protein A/G. Cross-linking was reversed by overnight incubation (70 °C) in TE buffer with 0.02% SDS, and chromatin was phenol/chloroform extracted. Anti-AID antibodies were kindly provided by Dr P. Gearhart. Anti-H3K4me3 was obtained from Millipore (ref: 07473), and anti-K3K27ac and anti-P-ser5 pol II pause were obtained from Abcam (clone ab4729 and ab5131, respectively). PCR primers are detailed in Supplementary Table 1.

### *IgH* primary transcription analysis

Total RNA was phenol–chloroform-extracted from 5 × 10^5^ B cells from freshly isolated peyers patches, in mice previously stimulated, as described above for SHM analysis. Real-time PCR was performed in duplicate by using TaqMan assay reagents and analysed on a StepOnePlus RT PCR system (Applied Biosystems). *IgH* primary transcripts (probe located in the intron between the last *J*_*H*_ and the intronic *Eμ* enhancer) were studied as previously reported^37^. Forward primer, 5′-TTCTGAGCATTGCAGACTAATCTTG-3′; reverse primer, 5′-CCTAGACAGTTTATTTCCCAACTTCTC-3′; and probe, 5′-CCCTGAGGGAGCCG-3′. κ-primary transcripts (probe located in the intron between the last *Jκ* and the intronic enhancer *Ei* κ): κ-forward primer, 5′-ACCCCCGCGGTAGCA-3′; κ-reverse primer, 5′-TCCTATCACTGTGCCTCAGGAA-3′; and probe, 5′-CCCTTGCTCCGCGTGGACCA-3′. Aicda transcripts were also analysed (reference Mm01184115-m1) and GAPDH was used for the normalisation of gene expression levels (reference Mm99999915-g1).

### Sequencing

Mice were immunised orally with sheep red blood cells for 2 weeks and intraperitoneally with 10 μg of LPS for 3 days. Single-cell suspensions from Peyer's patches were labelled with B220-APC- (Biolegend, ref:103212), GL7-FITC- (Beckton Dikinson, ref: 561530) and Fas-PE- (Beckton Dikinson, ref: 554258) conjugated antibodies. Purification of B220^+^GL7^+^Fas^+^ cells was realized on a FACS ARIA III (BD). Genomic DNA was extracted, and a region corresponding to a sequence of 517 bp downstream of the *J*_*H4*_ segment was amplified by PCR. As a control, *Igκ* light-chain *VJ*-rearranged fragments were also amplified. Primers (detailed in the Supplementary Table 1) were coupled to 454 Sequencing adaptor sequences and PCR was performed using the program previously reported^8^. According to the manufacturer, the resulting purified amplicons were prepared for sequencing with a GS Junior Titanium emPCR Kit (Lib-A; Roche), and the library of DNA fragments was sequenced on a 454 GS Junior instrument (Roche). Obtained sequences were aligned to the reference sequence using BWA aligner^38^, and SAMtools software was used to obtain BAM files^39^. Redundant sequences were excluded, and wig files were generated using IGV Tools^40^ and manually analysed to determine mutation frequencies for each nucleotide in the sequence.

### RNAseq experiments

CD43^−^ splenocytes were obtained from 4 *wt*, 4 ΔleftPAL and 4 Δ*IRIS* mice before and after 48 h of *in vitro* stimulation (1 × 10^6^ cells per ml in RPMI 1640 with 10% fetal calf serum) with 5μg ml^−1^ LPS. RNA was extracted using the miRNeasy kit from QIAGEN, according to the manufacturer's instructions. Two pooled RNAs (with two samples) were obtained for each genotype. RNA libraries were obtained using TruSeq Stranded Total RNA with Ribo-Zero Gold (Illumina), according to the manufacturer's instruction. Libraries were sequenced on a NextSeq500 sequencer, using NextSeq 500/550 High Output Kit (Illumina). Illumina NextSeq500 paired-end 2 × 150-nt reads were mapped with STAR release v2.4.0a versus mm10 with gene model from Ensembl release 77 with default parameters. Quantification of genes was then performed using feature Counts release subread-1.4.6-p1-Linux-x86_64 with ‘--primary -g gene_name -p -s 1 -M ' options based on Ensembl GTF release 77 annotations.

## Additional information

**Accession codes:** The RNA-seq data derived from the CD43^−^ splenocytes has been deposited at the GEO under the accession code GSE76359.

**How to cite this article:** Saintamand, A. *et al*. Deciphering the importance of the palindromic architecture of the immunoglobulin heavy chain 3' regulatory region. *Nat. Commun.* 7:10730 doi: 10.1038/ncomms10730 (2016).

## Supplementary Material

Supplementary Information Supplementary Figure 1, Supplementary Table 1 and Supplementary References

## References

[b1] Henderson A. & Calame K. Transcription regulation during B cell development. Annu. Rev. Immunol. 16, 163–200 (1998). 9597128 10.1146/annurev.immunol.16.1.163

[b2] Pinaud E. . The IgH locus 3' regulatory region: pulling the strings from behind. Adv. Immunol. 110, 27–70 (2011). 21762815 10.1016/B978-0-12-387663-8.00002-8

[b3] Perlot T., Alt F. W., Bassing C. H., Suh H. & Pinaud E. Elucidation of IgH intronic enhancer functions via germ-line deletion. Proc. Natl Acad. Sci. USA 42, 14362–14367 (2005). 16186486 10.1073/pnas.0507090102 PMC1242331

[b4] Marquet M. . The Eμ enhancer region influences H chain expression and B cell fate without impacting IgVH repertoire and immune response *in vivo*. J. Immunol. 193, 1171–1183 (2014). 24965776 10.4049/jimmunol.1302868

[b5] Saintamand A. . The IgH 3' regulatory region governs μ chain transcription in mature B lymphocytes and the B cell fate. Oncotarget 6, 4845–4852 (2015). 25742787 10.18632/oncotarget.3010 PMC4467119

[b6] Vincent-Fabert C. . Genomic deletion of the whole IgH 3' regulatory region (hs3a, hs1,2, hs3b, hs4) dramatically affects class switch recombination and Ig secretion to all isotypes. Blood 116, 1895–1898 (2010). 20538806 10.1182/blood-2010-01-264689

[b7] Rouaud P. . Elucidation of the enigmatic IgD class switch recombination via germ-line deletion of the IgH 3' regulatory region. J. Exp. Med. 211, 975–985 (2014). 24752300 10.1084/jem.20131385 PMC4010897

[b8] Péron S. . AID-driven deletion causes immunoglobulin heavy chain ‘locus suicide recombination' in B cells. Science 336, 931–934 (2012). 22539552 10.1126/science.1218692

[b9] Rouaud P. . The IgH 3' regulatory region controls AID-induced somatic hypermutation in germinal centre B-cells in mice. J. Exp. Med. 210, 1501–1507 (2013). 23825188 10.1084/jem.20130072 PMC3727322

[b10] Rouaud P. . Enhancers located in heavy chain regulatory region (hs3a, hs1,2, hs3b and hs4) are dispensable for diversity of VDJ recombination. J. Biol. Chem. 287, 8356–8360 (2012). 22270371 10.1074/jbc.M112.341024 PMC3318744

[b11] Medvedovic J. . Flexible long-range loops in the VH gene region of the Igh locus that likely facilitate the generation of a diverse antibody repertoire. Immunity 39, 229–244 (2013). 23973221 10.1016/j.immuni.2013.08.011 PMC4810778

[b12] Braikia F. Z. . A developmental switch in the transcriptional activity of a long-range regulatory element. Mol. Cell. Biol. 35, 3370–3380 (2015). 26195822 10.1128/MCB.00509-15 PMC4561733

[b13] Chauveau C. & Cogné M. Palindromic structure of the IgH 3' locus control region. Nat. Genet. 14, 15–16 (1996). 8782813 10.1038/ng0996-15

[b14] Chauveau C., Decourt C. & Cogné M. Insertion of the IgH locus 3' regulatory palindrome in expression vectors warrants sure and efficient expression in stable B cell transfectants. Gene 222, 279–285 (1998). 9831663 10.1016/s0378-1119(98)00475-2

[b15] D'addabbo P., Scascitelli M., Giambra V., Rocchi M. & Frezza D. Position and sequence conservation in Amniota of polymorphic enhancer HS1,2 whithin the palindrome of IgH 3' regulatory region. BMC Evol. Biol. 11, 71 (2011). 21406099 10.1186/1471-2148-11-71 PMC3068965

[b16] Kitabatake M. . Transgenic overexpression of G5PR that is normally augmented in centrocytes impairs the enrichment of high-affinity antigen-specific B cells, increases peritoneal B-1a cells, and induces autoimmunity in aged female mice. J. Immunol. 189, 1193–1201 (2012). 22753944 10.4049/jimmunol.1102774

[b17] Fukita Y., Jacobs H. & Rajewsky K. Somatic hypermutation in the heavy chain locus correlates with transcription. Immunity 9, 105–114 (1998). 9697840 10.1016/s1074-7613(00)80592-0

[b18] Le Morvan C., Pinaud E., Decourt C., Cuvillier A. & Cogné M. The immunoglobulin heavy-chain locus hs3b and hs4 3' enhancers are dispensable for VDJ assembly and somatic hypermutation. Blood 102, 1421–1427 (2003). 12714490 10.1182/blood-2002-12-3827

[b19] Pinaud E. . Localization of the 3' IgH locus elements that effect long-distance regulation of class switch recombination. Immunity 15, 187–199 (2001). 11520455 10.1016/s1074-7613(01)00181-9

[b20] Saintamand A. . Elucidation of IgH 3' region regulatory role during class switch recombination via germ-line deletion. Nat. Commun. 6, 7084 (2015). 25959683 10.1038/ncomms8084

[b21] Pefanis E. . RNA exosome-regulated long non-coding RNA transcription controls super-enhancer activity. Cell 161, 774–789 (2015). 25957685 10.1016/j.cell.2015.04.034 PMC4428671

[b22] Stanlie A., Masatoshi A., Muramatsu M., Honjo T. & Begum N. A. Histone3 lysine4 trimethylation regulated by the facilitates chromatin transcription complex is critical for DNA cleavage in class switch recombination. Proc. Natl Acad. Sci. USA 107, 22190–22195 (2010). 21139053 10.1073/pnas.1016923108 PMC3009800

[b23] Daniel J. A. & Nessenzweig A. Roles for histone H3K4 methyltransferase activities during immunoglobulin class-switch recombination. Biochim. Biophys. Acta 1819, 733–738 (2012). 22710321 10.1016/j.bbagrm.2012.01.019 PMC3378979

[b24] Cogné M. . A class switch control region at the 3' end of the immunoglobulin heavy chain locus. Cell 77, 737–747 (1994). 8205622 10.1016/0092-8674(94)90057-4

[b25] Manis J. P. . Class switching in B cells lacking 3' immunoglobulin heavy chain enhancers. J. Exp. Med. 188, 1421–1431 (1998). 9782119 10.1084/jem.188.8.1421 PMC2213411

[b26] Bébin A. G. . *In vivo* redundant function of the 3' IgH regulatory element HS3b in the mouse. J. Immunol. 184, 3710–3717 (2010). 20176739 10.4049/jimmunol.0901978

[b27] Vincent-Fabert C. . Ig synthesis and class switching do not require the presence of the hs4 enhancer in the 3' IgH regulatory region. J. Immunol. 182, 6926–6932 (2009). 19454689 10.4049/jimmunol.0900214

[b28] Dickel D. E., Visel A. & Pennacchio L. A. Functional anatomy of distant-acting mammalian enhancers. Philos. Trans. R. Soc. Lond. B. Biol. Sci. 368, 20120359 (2013). 23650633 10.1098/rstb.2012.0359 PMC3682724

[b29] Zhang Z. Z. . The strength of an Ig switch region is determined by its ability to drive R loop formation and its number of WGCW sites. Cell Rep. 8, 557–569 (2014). 25017067 10.1016/j.celrep.2014.06.021 PMC4118936

[b30] Kato Y. P. . Detection and characterization of R-loops at the murine immunoglobulin Sα region. Mol. Immunol. 54, 208–216 (2013). 23287599 10.1016/j.molimm.2012.11.009

[b31] Cogné M. Activation-induced deaminase in B lymphocyte maturation and beyond. Biomed. J. 36, 259–268 (2013). 24385067 10.4103/2319-4170.113191

[b32] Ronai D. . Detection of chromatin-associated single-stranded DNA in regions targeted for somatic hypermutation. J. Exp. Med. 204, 181–190 (2007). 17227912 10.1084/jem.20062032 PMC2118410

[b33] Laffleur B. . AID-induced remodelling of immunoglobulin genes and B cell fate. Oncotarget 5, 1118–1131 (2014). 24851241 10.18632/oncotarget.1546 PMC4012742

[b34] Kato L. . An evolutionary view of the mechanism for immune and genome diversity. J. Immunol. 188, 3559–3566 (2012). 22492685 10.4049/jimmunol.1102397

[b35] Böhmdorfer G. & Wierzbicki A. T. Control of chromatin structure by long noncoding RNA. Trends. Cell. Biol. 25, 623–632 (2015). 26410408 10.1016/j.tcb.2015.07.002 PMC4584417

[b36] Truffinet V. . The 3' IgH locus control region is sufficient to deregulate a c-myc transgene and promote mature B cell malignancies with a predominant Burkitt-like phenotype. J. Immunol. 179, 6033–6042 (2007). 17947677 10.4049/jimmunol.179.9.6033

[b37] Tinguely A. . Cross talk between immunoglobulin heavy-chain transcription and RNA surveillance during B cell development. Mol. Cell. Biol. 32, 107–117 (2012). 22037763 10.1128/MCB.06138-11 PMC3255717

[b38] Li H. Aligning sequence reads, clone sequences and assembly contigs with BWA-MEM. Preprint at http://arxiv.org/pdf/1303.3997.pdf (2013).

[b39] Li H. . The Sequence alignment/map (SAM) format and SAMtools. Bioinformatics. 25, 2078–2079 (2009). 19505943 10.1093/bioinformatics/btp352 PMC2723002

[b40] Thorvaldsdóttir H., Robinson J. T. & Mesirov J. P. Integrative Genomics Viewer (IGV): high-performance genomics data visualization and exploration. Brief. Bioinform. 14, 178–192 (2013). 22517427 10.1093/bib/bbs017 PMC3603213

