# Supplementary material for: Deciphering the importance of the palindromic architecture of the immunoglobulin heavy-chain 3' regulatory region
Source: Nat Commun. 2016 Feb 17;7:10730. doi: 10.1038/ncomms10730 (PMC4757795; doi:10.1038/ncomms10730)
Supplement: Supplementary Information — Supplementary Figure 1, Supplementary Table 1 and Supplementary References [file ncomms10730-s1.pdf]

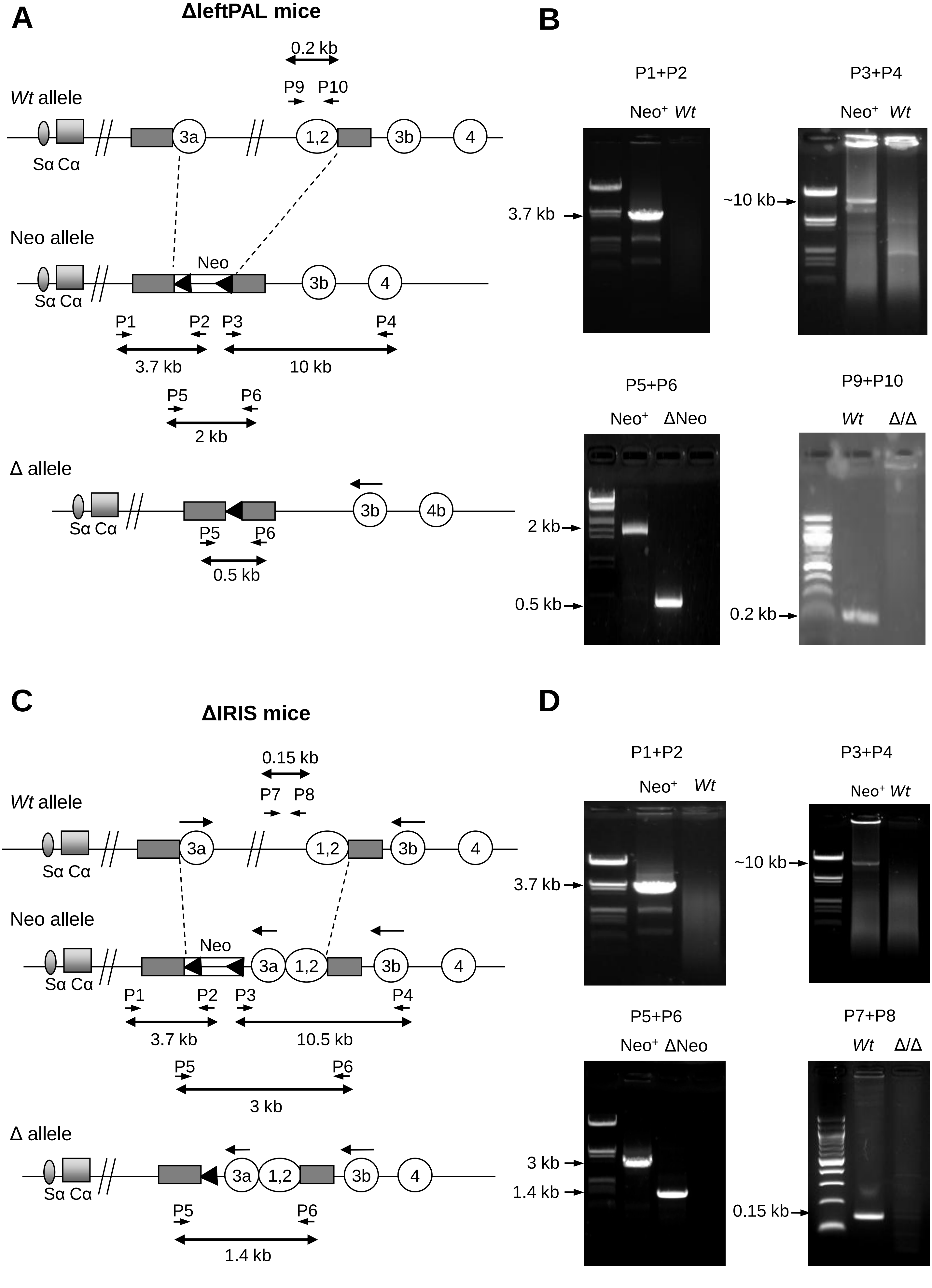

Supplementary Figure 1

Supplementary Figure 1 - Generation of ΔleftPAL and ΔIRIS mice. Upper part (A+B): generation of ΔleftPAL mice. A: Gene-targeting vector was constructed in order to replace the genomic fragment encompassing the hs3a+hs1,2 part of the 3'RR with a loxP/neoR gene cassette in ES cells. Mutant ES clones allowed the derivation of Neo mutant mice. Neo mutant mice allowed the derivation of ΔleftPAL animals after cre-deletion of the neo gene. B: A specific PCR was performed to ensure correct 5' recombination (P1+P2) (amplicon of 3.7 kb) in ES cells. A specific PCR was performed to ensure correct 3' recombination (P3+P4) (amplicon of 10 kb) in ES cells. A specific PCR was performed to ensure correct Cre recombination (P5+P6) (amplicon of 2 kb vs 0.5 kb after Cre deletion) in Neo mutant mice. A specific PCR was performed to ensure homozygous ΔleftPAL mice (P9+P10) (lack of a 0.2 kb amplicon). Lower part (C+D): Generation of ΔIRIS mice. C: Same protocol that in the upper part except that the loxP/neoR gene cassette reintroduced inverted hs3a and hs1,2 enhancers. D: A specific PCR was performed to ensure correct 5' recombination (P1+P2) (amplicon of 3.7 kb) in ES cells. A specific PCR was performed to ensure correct 3' recombination (P3+P4) (amplicon of 10 kb) in ES cells. A specific PCR was performed to ensure correct Cre recombination (P5+P6) (amplicon of 3 kb vs 1.4 kb after Cre deletion) in Neo mutant mice. A specific PCR was performed to ensure homozygous ΔIRIS mice (P7+P8) (lack of a 0.15 kb amplicon).

Screening

P1	5'-GGA GGG AGA AAT ACC ACC-3'	
P2	5’ AGC ACG TAC TCG GAT GGA AG 3’	
P3	5’GTG CAA TCC ATC TTG TTC AAT GGC CGA TCC 3’	
P4	5’ CGG CTC TAA CAA CTG GGT CCT GTA C 3’	
P5	5'-GGT TAA TCA GTT GGG GCT GA-3'	
P6	5'-AGG TGC ATG ACA GGG TAA GG-3'	
P7	5'-GGT GAC ATT AAG CTT GGG GAT A-3'	
P8	5'-CCC TGA GAG GTT TCA CAT TTT C-3'	
P9	5'-ACC ACT GCT GGA ATC TGA CC-3'	
P10	5'-CAG GCC AAG ACT TTT CCA GT-3'	

SHM analysis

VHJ558 SHM	5'-CAG CCT GAC ATC TGA GGA CTC TGC-3'	
SHM H4 3'	5'-CAG CAA CTA CCC TTT TGA GAC CGA-3'	

ChIP analysis

ChIP A Fw	5'-CAG TCT CCT CAG GTG AGT CCT-3'	Maul et al, 2014
ChIP A Rev	5'-CCC AAT GAC CCT TTC TGA CT-3'	
ChIP B Fw	5'-GGA GAG CTG TCT TAG TGA TTG AGT CAA GGG-3'	
ChIP B Rev	5'-GTG TTC CTT TGA AAG CTG GAC-3'	
Sμ-U Fw	5'-TCTAAAATGCGCTAAACTGAGG-3'	Wang et al., 2009
Sμ-U Rev	5'-AGCGTAGCATAGCTGAGCTC-3'	
Iγ3 Fw	5'-AGATCCCAAAGCTAAGCTCCTG-3'	Wang et al., 2006
Iγ3 Rev	5'-CAGAGAGACCCCCTCCACAGT-3'	
Sγ3-U Fw	5'-GCTGAGAGTATGCACAGCCA-3'	
Sγ3-U Rev	5'-GGATCATGGAAACTCCTCCG-3'	
Sγ3-D Fw	5'-CAGGCTGGGAAACTCTTGGG-3'	
Sγ3-D Rev	5'-AGGTTAGTCCCCATACTTGAACC-3'	
Iγ2b Fw	5'-TATTGTTCTGGCTGCAAATGG-3'	Li et al, 2013
Iγ2b Rev	5'-GTGCAGGCATATCTTCCATCC-3'	
Sγ2b-U Fw	5'-AGCTCCAAAAGCTCAGCAGAC-3'	
Sγ2b-U Rev	5'-AGCCCCAGCTTACAAAGAGCT-3'	
Sγ2b-D Fw	5'-GGTGGGAATATGAGGGAGAAGTCCTAG-3'	
Sγ2b-D Rev	5'-TTCCACCTGCCTCAGCTCTCCCACAGC-3'	

GLT Analysis

Iμ-Cμ-Fwd	5'-ACCTGGGAATGTATGGTTGTGGCTT-3'	Park et al, 2009
Iμ-Cμ-Rev	5'-TCTGAACCTTCAAGGATGCTCTTG-3'	
Iγ3-Cγ3-Fwd	5'-AACTACTGCTACCACCACCACCAG-3'	
Iγ3-Cγ3-Rev	5'-ACCAAGGGATAGACAGATGGGG-3'	
Iγ1-Cγ1-Fwd	5'-GGCCCTTCCAGATCTTTGAG-3'	
Iγ1-Cγ1-Rev	5'-ATGGAGTTAGTTTGGGCAGCA-3'	
Iγ2b-Cγ2b-Fwd	5'-CCAACCAGGAAGAGTCCAGAG-3'	
Iγ2b-Cγ2b-Rev	5'-ACAGGGATCCAGAGTTCCAAGT-3'	
IGγa-Cγ2a-Fwd	5'-GCTGATGTACCTACCGAGAGA-3'	
Iγ2a-Cγ2a-Rev	5'-GCTGGGCCAGGTGCTCGAGGTT-3'	
Iα-Cα Fw	5'-CTACCATAGGGAAGATAGCCT-3'	Park et al, 2005
Iα-Cα-Rev	5'-TAATCGTGAATCAGGCAG-3'	

Supplementary table 1 - PCR primers used for the screening of ΔleftPAL and ΔIRIS mice, for real-time PCR analysis and sequencing

Supplementary References

Li, G. et *al.* Combinatorial H3K9acS10ph Histone Modifications in *IgH* Locus S Regions Target 14-3-3 Adaptors and AID to Specify Antibody Class Switch DNA Recombination. *Cell Rep*. **5(3)**: 702–714. (2013)

Maul, R. et *al.* Spt5 accumulation at variable genes distinguishes somatic hypermutation in germinal center B cells from ex vivo–activated cells. *J. Exp. Med.* **211**:2297-2306 (2014)

Park, SR. et *al.* Analysis of transforming growth factor-beta1-induced Ig germ-line gamma2b transcription and its implication for IgA isotype switching. *Eur J Immunol.* **35(3)**:946-56. (2005)

Park, SR. et *al.* HoxC4 binds to the promoter of the cytidine deaminase AID gene to induce AID expression, class-switch DNA recombination and somatic hypermutation. *Nature Immunology* **10**, 540 - 550 (2009)

Wang, L. et *al.* AID-dependent histone acetylation is detected in immunoglobulin S regions. *J Exp Med.* **203(1)**:215-26. (2006)

Wang, L. et *al.* S region sequence, RNA polymerase II, and histone modifications create chromatin accessibility during class switch recombination. *J. Exp. Med.* **206**:1817-1830 (2009)
